# Through their eyes: Multi-subject brain decoding with simple alignment techniques

**DOI:** 10.1162/imag_a_00170

**Published:** 2024-05-08

**Authors:** Matteo Ferrante, Tommaso Boccato, Furkan Ozcelik, Rufin VanRullen, Nicola Toschi

**Affiliations:** Department of Biomedicine and Prevention, University of Rome Tor Vergata, Rome, Italy; CerCo, CNRS UMR5549, Toulouse, France; Universite de Toulouse, Toulouse, France; ANITI, Toulouse, France; Martinos Center For Biomedical Imaging, MGH and Harvard Medical School, Boston, MA, United States

**Keywords:** brain decoding, neuroscience, cross subject decoding

## Abstract

To-date, brain decoding literature has focused on single-subject studies, that is, reconstructing stimuli presented to a subject under fMRI acquisition from the fMRI activity of the same subject. The objective of this study is to introduce a generalization technique that enables the decoding of a subject’s brain based on fMRI activity of another subject, that is, cross-subject brain decoding. To this end, we also explore cross-subject data alignment techniques. Data alignment is the attempt to register different subjects in a common anatomical or functional space for further and more general analysis.

We utilized the Natural Scenes Dataset, a comprehensive 7T fMRI experiment focused on vision of natural images. The dataset contains fMRI data from multiple subjects exposed to 9,841 images, where 982 images have been viewed by all subjects. Our method involved training a decoding model on one subject’s data, aligning new data from other subjects to this space, and testing the decoding on the second subject based on information aligned to the first subject. We also compared different techniques for fMRI data alignment, specifically ridge regression, hyper alignment, and anatomical alignment.

We found that cross-subject brain decoding is possible, even with a small subset of the dataset, specifically, using the common data, which are around10%of the total data, namely 982 images, with performances in decoding comparable to the ones achieved by single-subject decoding. Cross-subject decoding is still feasible using half or a quarter of this number of images with slightly lower performances. Ridge regression emerged as the best method for functional alignment in fine-grained information decoding, outperforming all other techniques.

By aligning multiple subjects, we achieved high-quality brain decoding and a potential reduction in scan time by90%. This substantial decrease in scan time could open up unprecedented opportunities for more efficient experiment execution and further advancements in the field, which commonly requires prohibitive (20 hours) scan time per subject.

## 1 Introduction

Deep learning has revolutionized numerous fields, including neuroscience (Badrulhisham et al., 2024;Lange et al., 2023;Richards et al., 2019;Vu et al., 2018). The application of deep learning techniques in neuroscience has led to significant advancements in understanding brain function and decoding the intricate workings of the human mind (Oota et al., 2023). Brain decoding, in particular, has emerged as a crucial area where deep learning plays a pivotal role.

Brain decoding involves the extraction of meaningful information from recorded brain activity, allowing researchers to infer mental states, perceptual experiences, or cognitive processes. For example, deep learning algorithms have been used to decode brain activity and predict whether an individual is looking at a face or an object based on their neural responses (Awangga et al., 2020;Oota et al., 2023;Zafar et al., 2015).

The potential applications of brain decoding are vast, from understanding various aspects of brain function, such as information processing strategies, decision making, memory formation, and consolidation, to potential uses in neurofeedback, neuroaesthetics, or neuromarketing strategies (Du et al., 2022). Moreover, successful brain decoding could lead to novel strategies for diagnosing and treating neurological or neuropsychiatric conditions, and potentially contribute to the development of radically new algorithmic learning strategies. However, these promising endeavors are not without challenges. Noninvasive data, for instance, have lower temporal or spatial resolution than neural firing, which may limit the granularity of information that can be retrieved. Furthermore, physiological noise and signal/image artifacts can affect both fMRI and EEG data, which can only be imperfectly removed after the data are acquired. Nevertheless, several brain decoding studies have achieved impressive results (Awangga et al., 2020;Zafar et al., 2015).

One of the key challenges in brain decoding is the subject-specific nature of all models developed thus far. This means that the models are tailored to individual subjects, which can lead to significant variability in the results, given that the amount of data collection per subject could be limited by external factors like time and acquisition costs. Moreover, intrinsic inter-individual variability poses further challenges and every model has to be built from scratch for each new subject. This variability is a consequence of the unique functional and anatomical structure of each individual’s brain, and implies the need to acquire an entire dataset and train an individual model for each subject. This technology’s use is limited by a bottleneck requiring extensive data collection—typically thousands of stimulus images—to function properly. This complexity stems from the unique brain anatomy, information processing methods, and functional responses each individual possesses, complicating the training of a universally applicable brain activity decoding model. Despite individual differences in brain anatomy and function, common structures enable reliable neuroscience analysis using template matching techniques, such as anatomical and functional alignment. Anatomical alignment transforms individual brain images to match a standard “average” brain template or “atlas,” aligning the size, shape, and orientation of brain images. This facilitates meaningful cross-comparisons of brain images, although it is more effective for larger, well-defined structures and may lack precision for smaller, variable regions. Additionally, it does not account for functional differences across brains. Thus, anatomical alignment is often supplemented with functional alignment, which synchronizes brain activity patterns across individuals, aiding the comparison and analysis of functional data. This method is vital as activity locations can differ among individuals. Numerous functional alignment methods exist, each with distinct applications and limitations.

In this study, we explore and contrast three methods for cross-subject brain decoding of visual stimuli, using the established, cutting-edge Brain-Diffuser decoding procedure. This approach mitigates variability from new decoding procedures, enabling straightforward quantitative and qualitative comparisons of cross-subject decoding outcomes.

**Fig. 1.**
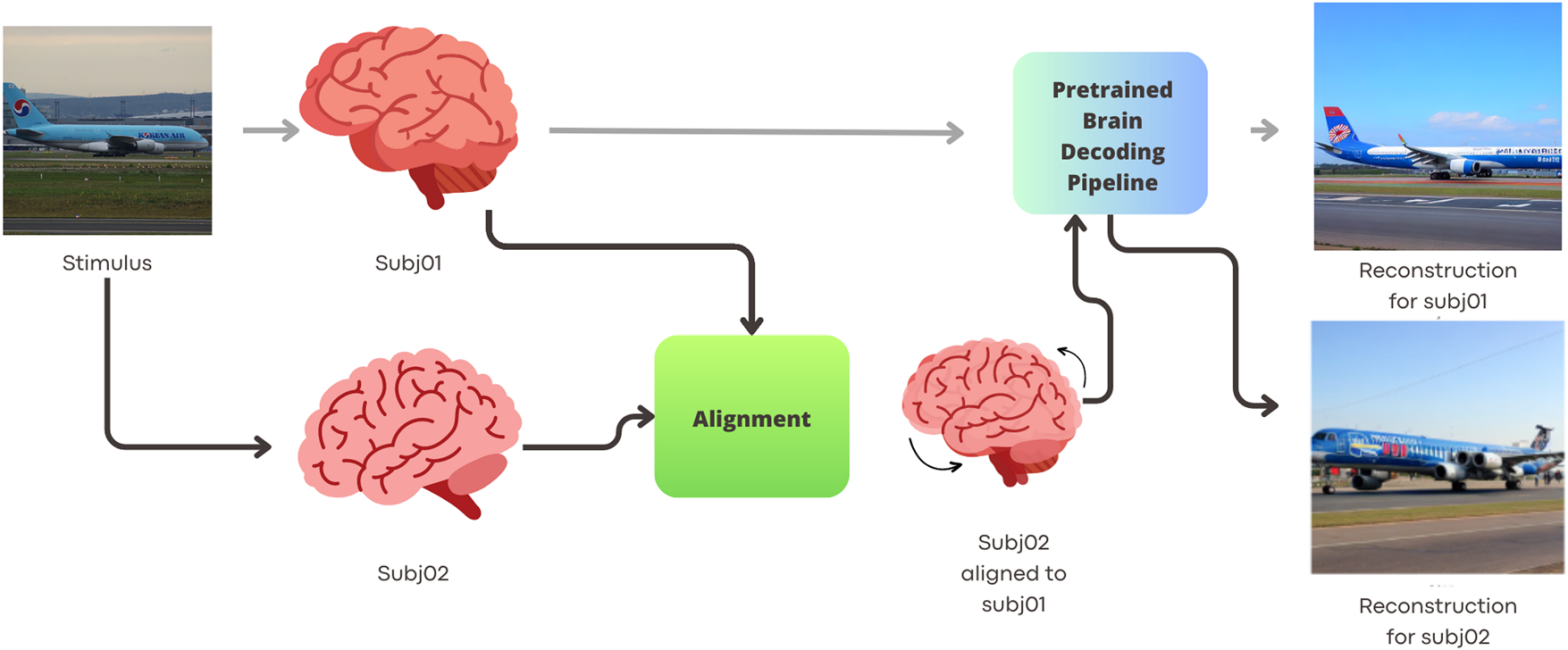
Scheme for cross-subject decoding: The procedure involves the following steps: In the first step, Subj01 (top row) is selected as the target subject. A decoding model is trained to reconstruct seen images based on the brain activity of Subj01 on the training set of Subj01 images (8,859 images). These images are shown only to this subject. Next, we decode the brain activity of a second subject, Subj02, who was exposed to a share of the same stimuli that Subj01 was exposed to (982 images). Using the shared images, we can compare the brain activity related to the same stimuli across different subjects. We used this shared information to align the functional activity of Subj02 with that of the target subject. Once the alignment transformation is learned, we can align the complete dataset (including data not used for alignment nor for decoder training) and we can utilize the pretrained decoder to reconstruct images from Subj02 without training a decoding model specifically for Subj02.

We train a visual stimuli decoding model on one subject (Subj01 usually used as template, except in comparison between target subject section) and employ anatomical alignment, hyper alignment, and functional alignment with ridge regression for three others, decoding their activity using the pretrained model. Our goal is to demonstrate the feasibility of fine-grained cross-subject decoding for visual stimuli reconstruction, potentially reducing scan times significantly by only acquiring data necessary for alignment, thereby reaching state-of-the-art in image reconstruction.Figure 1outlines our proposed pipeline, whileFigure 2provides examples of cross-subject decoding results.

**Fig. 2.**
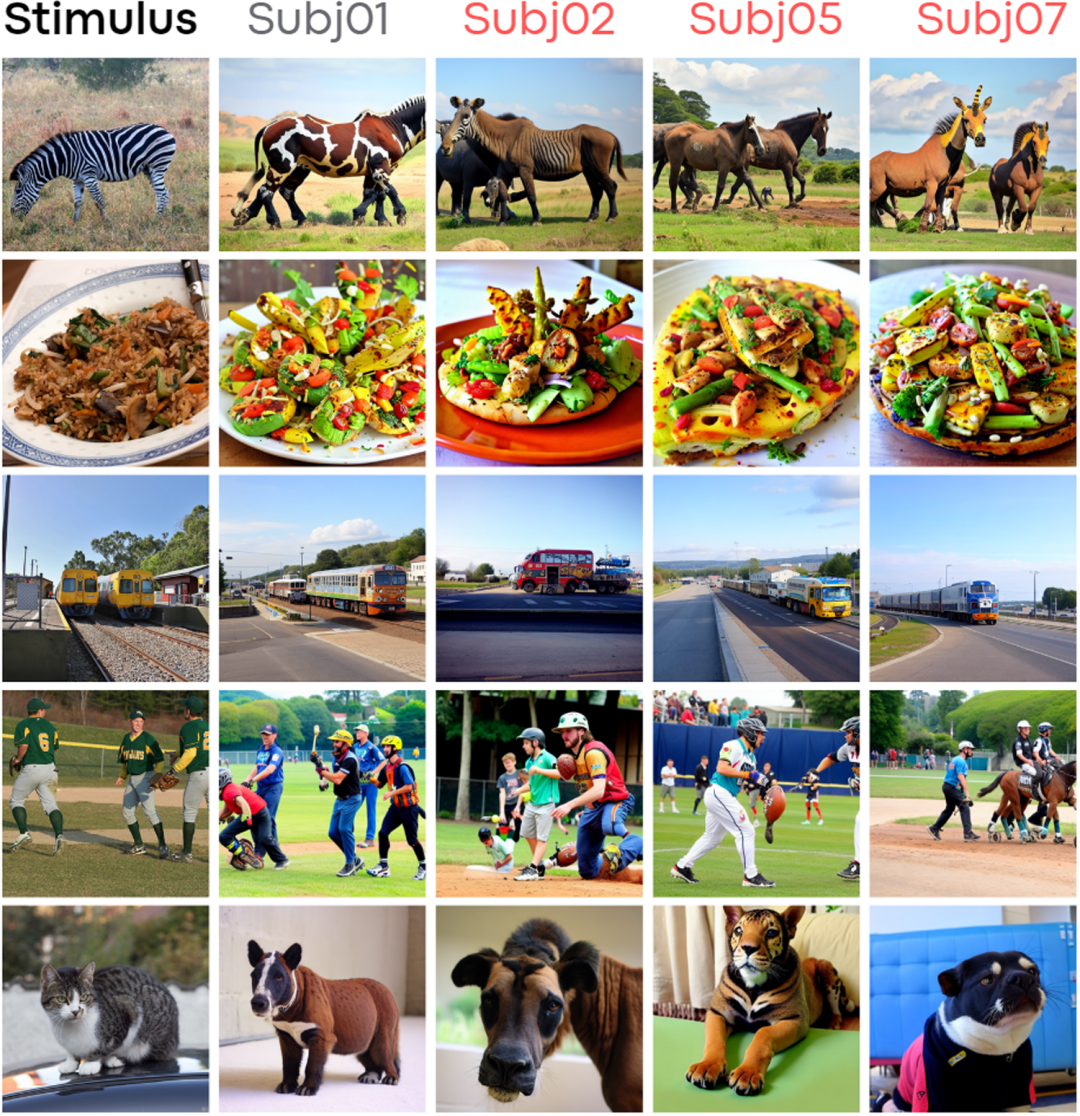
Example results: The first column, “Stimulus,” presents the stimuli from the fMRI experiment. The “Subj01” column (in gray) displays the decoded activity from Subj01, providing an upper performance baseline using a subject-specific decoder model. All other columns (in red) show results from functional alignment using Ridge Regression with100%common data (952 images), meaning subjects were functionally aligned to Subj01 and decoded using Subj01’s trained decoder. To ensure robust visual comparisons, none of the displayed images were used in learning alignment transformation, demonstrating functional alignment on unseen data not used for decoder training or alignment function learning.

## 2 Related Work

In the evolving field of deep learning-based brain decoding, a range of models has been utilized to scrutinize preprocessed fMRI time series as input, particularly focusing on visual stimuli decoding. This involves reconstructing images that could have triggered specific fMRI patterns—termed brain activity. Here, we review major works in this domain. Some methods have employed variational autoencoders with a generative adversarial component (VAE-GAN) to encode latent human face representations, estimating these encoded representations from fMRI activity using a linear model (VanRullen & Reddy, 2019). Sparse linear regression has also been utilized on preprocessed fMRI data to predict features from the early convolutional layers of a pre-trained convolutional neural network (CNN) for natural images (Horikawa & Kamitani, 2017). Unsupervised and adversarial strategies have been used for image reconstruction, including dual VAEGAN and unsupervised methods for decoding fMRI stimuli, utilizing multiple encoder and decoder approaches (Gaziv et al., 2022;Ren et al., 2019;Shen et al., 2019). Pretrained architectures like BigBiGAN and IC-GAN have optimized latent spaces, significantly enhancing high-fidelity image reconstruction from fMRI patterns (Casanova et al., 2021;Donahue & Simonyan, 2019). Recently, diffusion models have become prominent in the decoding pipeline, providing superior image generation performance (Takagi & Nishimoto, 2023;Z. Chen et al., 2022). These models often incorporate semantic-based strategies (Ferrante, Boccato, & Toschi, 2023) and multi-step decoding strategies (Ferrante, Boccato, Ozcelik, et al., 2023;Ozcelik & VanRullen, 2023).

Interesting approaches also include MindEye (Scotti et al., 2023), a reconstruction tool that effectively maps brain activity to multimodal latent spaces with a contrastive approach for accurate image retrieval and reconstruction, and DREAM (Xia et al., 2023) that reconstructs images from brain activities using fMRI, closely mirroring the human visual system’s structure. In general, the field of bidirectional brain computer interface research has opened avenues for diverse and innovative studies aimed at mapping inputs from and to the brain. Notable examples of such groundbreaking work include those byMai and Zhang (2023),Luo et al. (2023), andLiu et al. (2023), among others. Additionally, this research domain has expanded to encompass a variety of stimuli beyond traditional inputs. The Mind-Video study byZ. Chen et al. (2023)tackles the task of decoding video content from fMRI data. Moreover, a particularly captivating branch of research is dedicated to unraveling language processing within the brain through fMRI data, focusing on both the encoding of language into brain activity and the decoding of brain activity back into language. This dual approach has seen significant recent contributions, as evidenced by works such as those byCaucheteux and King (2022),Caucheteux, Gramfort, et al. (2022),Tang et al. (2023),Antonello et al. (2023), andHuth et al. (2012), marking crucial advancements in understanding and interpreting the neural substrates of language. Decoding language with non-invasive measurements has also been extended to other modalities, including MEG and EEG (Défossez et al., 2023;Duan et al., 2023;Wang and Ji, 2022), yielding promising results. Recent work fromBenchetrit et al. (2023)is specifically related because the architecture proposed to decode MEG signals includes an alignment module trained specifically for each subject. This is an elegant solution to train a model across subjects taking into consideration individual differences. In contrast to these approaches, in this paper, we explore avenues to leverage pretrained single-subject models with a separate alignment procedure, making it more flexible and agnostic to the specific decoding pipeline used. Finally, a recent survey byOota et al. (2023)compiles numerous works on encoding and decoding across different modalities, offering a comprehensive review for interested readers. Notably, most advanced image decoding techniques from fMRI (which is the focus of this study) employ a subject-specific approach requiring training or fine-tuning on individual subject data. Our main aim was therefore to enhance this approach by proposing a universal method that leverages functional alignment of neural data.

Regarding alignment techniques, there are several approaches (Bazeille et al., 2019,^2021^). Hyperalignment (Busch et al., 2021;Haxby et al., 2011,^2020^) aligns functional brain activity across individuals in a high-dimensional space, enhancing the precision of cross-subject brain activity predictions, but requires extensive high-quality data and complex computational resources. The Shared Response Model (SRM) (P.-H. C. Chen et al., 2015;H. Richard et al., 2019) aligns brain activity by identifying a common response pattern across subjects, ideal for shared experiences, but assumes uniform responses, which individual perception and cognition differences may contradict. Independent Component Analysis (ICA) (Calhoun et al., 2009) separates multivariate signals into additive subcomponents, identifying common brain activity patterns, but requires statistical independence of subcomponents, which may not always apply to brain data.

While functional alignment methods provide powerful tools for comparing brain activity, their limitations and assumptions require careful result interpretation. These methods align and compare functional brain data, complementing, not replacing, anatomical alignment. Various other methods, each with its pros and cons, have been proposed. The work most similar to ours in addressing decoding through functional alignment isHo et al. (2023), which demonstrates the potential for cross-subject decoding using linear regression between subjects. Our study contrasts anatomical alignment, hyperalignment-based functional alignment, and ridge regression-based alignment methods for cross-subject brain decoding across various dataset sizes. We specifically investigate the feasibility of cross-dataset decoding using a high-quality 7T dataset, offering robust evidence that simple methods can achieve high performance in cross-subject decoding.

## 3 Material and Methods

In this section, we describe the proposed method and the data we used. The data are publicly available and can be requested athttps://naturalscenesdataset.org/. All experiments and models were trained on a server equipped with four NVIDIA A100 GPU cards (80GB RAM each connected through NVLINK) and 2TB of System RAM.

The study utilizes the Natural Scenes Dataset (NSD) (Allen et al., 2022), a vast fMRI data set from eight subjects exposed to images from the COCO21 dataset. We focused on four subjects, forming a unique training dataset of 8,859 images and 24,980 fMRI trials, and a common dataset of 982 images and 2,770 trials. To reduce spatial dimensionality, we applied a mask to the fMRI signal (resolution of 1.8 mm isotropic) using the NSDGeneral ROI, targeting various visual areas. This strategic ROI selection enhanced the signal-to-noise ratio and simplified data complexity, enabling exploration of both low-level and high-level visual features. Temporal dimensionality was reduced using precomputed betas from a general linear model (GLM) (Kay et al., 2013;Prince et al., 2022)) with a fitted hemodynamic response function (HRF) and a denoising process as detailed in the NSD paper. Data from Subj01, Subj02, Subj05, Subj07, warped into the Montreal Neurological Institute common space (MNI) and downsampled at 2 mm, represented the brain activity of each subject and helped decrease computational time and cost. We used the common dataset as alignment, keeping out 30 images for visual comparison, so there are 8,859 unique images for each subject. We only used them for training the decoding model for Subj01. Then, there are 952 common images across all subjects that were used to functionally align them to the activity of Subj01, and 30 common images kept out for visual comparison on images neither used in the training nor in the alignment procedure. These 30 images were chosen because they are used as visual qualitative evaluation of decoding results in other papers and could help the reader to compare results across different methods. Decoding metrics are evaluated on the 952 images which correspond to our test set for each one of the subjects; since these images are never seen by the decoder model, so the evaluation is still fair and on unseen images. When we refer to100%of common data, we are pointing to these 952 images.

### 3.1 Decoding pipeline: Brain-diffuser

The “Brain-Diffuser” (Ozcelik & VanRullen, 2023) model is a two-stage framework for reconstructing natural scenes from fMRI signals. Initially, a Very Deep Variational Autoencoder (VDVAE) provides an “initial guess” of the reconstruction, focusing on low-level details. This guess is refined using high-level semantic features from CLIP-Text and CLIP-Vision models, employing a latent diffusion model (Versatile Diffusion) for final image generation. The model, represented inFigure 3, takes fMRI signals as input and generates reconstructed images, capturing low-level properties and overall layout. As a state-of-the-art procedure, Brain-Diffuser was trained using data from Subj01 in the MNI space (cross-subject decoding requires of a common space). Further details about the decoding model are available in the original paper. Aside from the choice of this specific pipeline, our method is universally applicable and can enhance any single-subject decoding pipeline. It serves as a versatile tool that can seamlessly integrate with new, cutting-edge pipelines. By focusing solely on preprocessing the input data, our approach allows the underlying pipeline, regardless of its specifics, to function effectively with single-subject fMRI data, thereby generating images without needing direct modifications to the pipeline itself. This “plug and play” capability ensures our method remains adaptable and effective in evolving research landscapes.

**Fig. 3.**
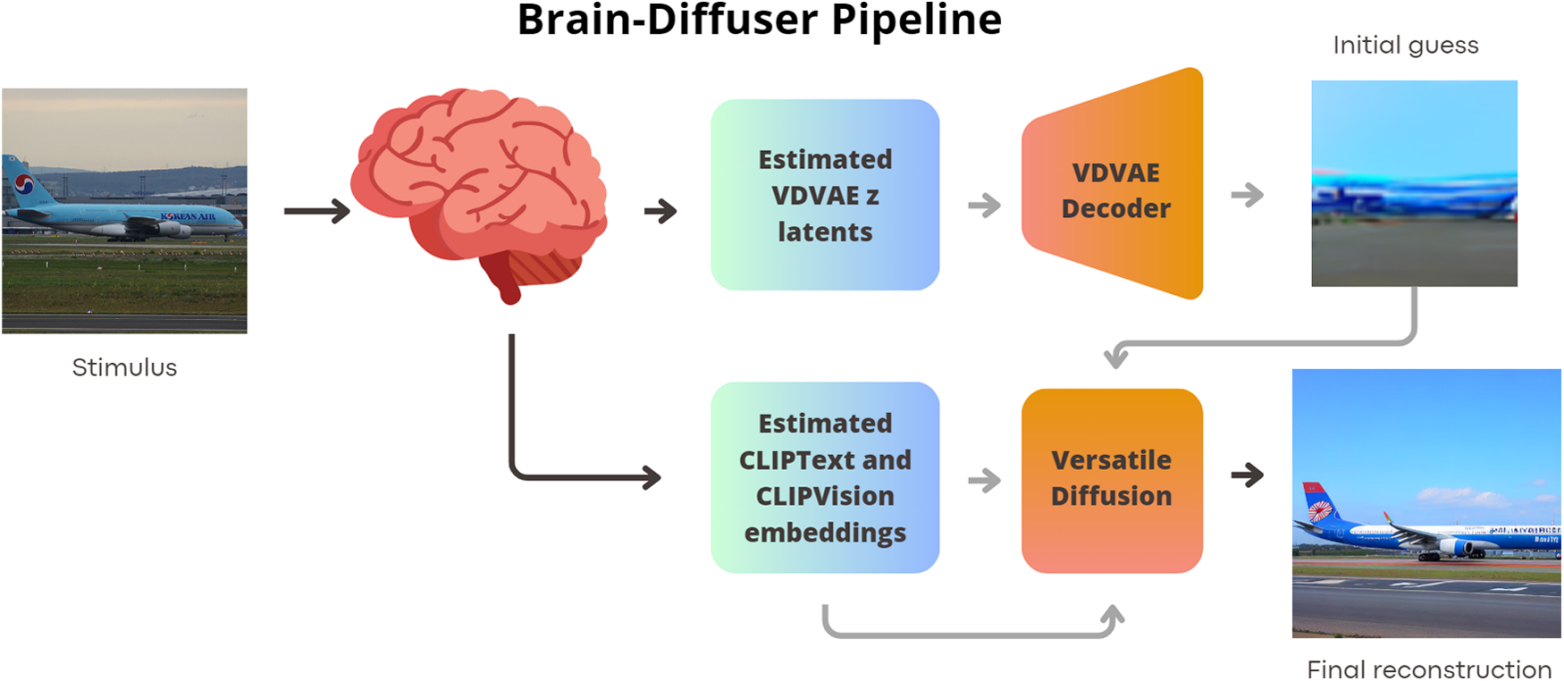
The Brain-Diffuser pipeline, the decoder for brain activity used in this study, begins with brain activity from viewing an image stimulus. A model is trained to estimate the latent representation of the VDVAE autoencoder as well as the text and visual embeddings of the CLIP model, using linear models. These estimated vectors and an initial guess image obtained by decoding the autoencoder latents are fed into Versatile Diffusion—a latent diffusion model—to reconstruct the final image.

### 3.2 Alignment

This study investigates three alignment strategies to evaluate cross-subject fine-grained brain decoding’s feasibility: anatomical alignment, functional alignment via hyper alignment, and functional alignment through ridge regression.

Anatomical alignment, our baseline, relies solely on anatomical details, transforming functional aspects using pre-computed structural image transformations. On the other hand, functional alignment necessitates a more comprehensive approach. Consider the scenario where the brain activity of a source subjectSneeds to align with a target subjectT. These activities, responses to numerous stimuli, are matrices of shape*(#stimuli,*#* voxels)*. Given that subjects encounter several common stimuli (i.e., they view identical images in the fMRI scanner), we can divide the datasets intoT_common_,T_different_andS_common_,S_different_. Our goal is to leverage thecommondataset portion to learn a mapping fromStoT, aligning the entireSdataset with theTfunctional space. The NSD experiment’s structure, with separate training and test sets (the latter containing identical images for each subject), provides a common stimuli set for alignment purposes.

#### 3.2.1 Anatomical alignment

Anatomical alignment, a common neuroscience method, aligns to a standard template, here, the MNI space, facilitating anatomical structure comparison. This alignment typically involves a linear coregistration of anatomical images between native and common spaces, followed by a nonlinear warping to match common brain structures. Several software options like FSL and ANTs can perform this task. The NSDData authors (Allen et al., 2022) elaborate on this process in their released code, providing betas (i.e., coefficients obtained by regressing the stimulus waveform against the fMRI data) for all subjects in the MNI common space (1 mm). We downsampled these to 2 mm to approximate the resolution used in the original Brain-Diffuser decoding paper (1.8 mm) and to reduce spatial dimensionality.

#### 3.2.2 Hyperalignment

HyperAlignment (Haxby et al., 2011,^2020^), a functional data alignment technique, models functional data as high-dimensional points, with each voxel representing a dimension with betas ranging inℛ. This method, based on Procrustes Analysis (Gower, 1975), presents a high-dimensional model of the representational space in the human ventral temporal (VT) cortex, wherein dimensions are response-tuning functions common across individuals.

To perform the Procrustes analysis for functional brain alignment, we aim to find a rotation matrixRand a scale factorcsuch that the difference$|c\text{SR}-\text{T}{|}^{2}$is minimized.

This is achieved by computing the matrix product$\text{P}={\text{S}}_{common}^{T}{\text{T}}_{common}$, performing the singular value decomposition ofPto obtain left and right eigenvector matricesUandV, and computing$\text{R}=\text{U}{\text{V}}^{T}$and the scaling factor$c=\frac{trace\left({\text{T}}_{\text{common}}^{\;\;\;\;\;\;\;\;T}\;({\text{S}}_{\text{common}}R)\right)}{trace\left({\text{S}}_{common}^{T}{\text{S}}_{common}\right)}$. Finally, we can apply the matrixRand the scalingcto both common and non-common source data to align them with the target subject. We computed these values for Subj02, Subj05, and Subj07 as source subjects, using Subj01 as the target, to align all subjects to the functional space of the first one. For detailed mathematical proofs and other insights, please refer to the original articles (Gower, 1975;Haxby et al., 2011,^2020^).

#### 3.2.3 Ridge regression

Our third approach embraces a simple assumption: even in different subjects, all functional data contain the information for the same stimuli, albeit possibly spread across different voxels. This suggests that one subject’s activity (source) might be expressed as a linear combination of the activity of another subject (target) for the same stimuli. By deriving a linear combination for each voxel of the target from all possible voxels of the source, we can create a linear map from the source to the target, facilitating functional alignment. The target subject activity can be expressed ast i=∑jw jsj, wheret iis thei-th activity of the target voxel for each common dataset stimulus. Here,t irepresents thei-th column ofT common, expressed as a linear combination of allS commoncolumns. The challenge lies in finding the vector ofwvalues. When extended to all the target subject voxels, thewvector morphs into a square matrixW, each column of which contains weights to estimate one target subject voxel from a combination of source values. The objective can be redefined as minimizing$|\text{S}\;common\;{\text{W}}^{T}-\text{T}\;common{|}^{2}$.

We employed Ridge Regression from (Buitinck et al., 2013) to determine theWmatrix, conducting a 5-fold cross-validation to select the optimal hyper-parameterαfrom the values[0,1,10,1e2,1e3,1e4]. Our findings indicated thatα=1000yields superior performance, hence we adopted it as our final regularization parameter. We computed these values to align all subjects to the initial functional space for the sources Subj02, Subj05, and Subj07, and using Subj01 as the target.

### 3.3 Evaluation

Our research seeks to evaluate visual stimuli’s detailed brain decoding feasibility, scrutinizing the alignment methods and shared data ratio at play. We examined how the alignment performance fluctuates when the shared data makes up10%,25%,50%, and100%of the total common data (952 images).

Our shared dataset, or “alignment dataset,” comprises 982 images, all viewed by every subject. In order to allow visual comparison, we excluded 30 images from the original Brain-Diffuser paper. Thus, these excluded images neither influenced the training of the decoding pipeline nor the alignment process. The remaining 952 images serve as the shared dataset. We computed transformations for each alignment method (anatomical, hyperalignment, ridge regression) and shared dataset proportion, applying the linear transformation to the complete dataset. This procedure aligns the images with Subject 01’s functional space. We then used the pre-trained Brain-Diffuser pipeline for decoding the aligned fMRI activity and reconstructing the images. We assessed our image reconstruction process through both basic and advanced metrics, including PixCorr, SSIM, and 2-way accuracy in AlexNet, Inception, and CLIP latent spaces. This comprehensive evaluation approach allows us to benchmark our results against other brain decoding studies. However, the goal here is not merely comparison, but rather the examination of performance in relation to the shared data fraction and alignment method, given a fixed decoding pipeline, trained solely on Subj01 as a reference target. These metrics enable us to benchmark our results against prior studies in decoding research. However, it is important to note that they rely on an extensive pipeline comprising multiple components, as outlined previously. Given our focus on the input data for the decoding model, which employs a linear decoder within the CLIP space, we also employ a critical evaluation metric: the Direct CLIP 2-way accuracy, assessed directly on the regressed CLIP Classification (CLS) embeddings, that is also part of our decoding linear layer. We refer to this measure as “Direct CLIP” to differentiate it from previously mentioned metrics.

### 3.4 Cross-dataset decoding experiment

In order to evaluate the generalizability of our decoding pipeline, we undertook cross-dataset decoding between different fMRI datasets.

Firstly, we undertook cross-dataset decoding between the BOLD5000 (Chang et al., 2019) and the Natural Scenes Dataset (NSD). The BOLD5000 dataset comprises fMRI data from five subjects who viewed 5,000 images. These data were acquired at a field strength of 3T, providing inherently lower signal-to-noise ratio than the 7T data present in the NSD. Additionally, BOLD5000 encompasses a narrower range of semantic categories in comparison to the diverse stimuli of the NSD. The protocols between the two datasets also differ: the NSD employs a rapid-event related protocol where images are displayed for 2 seconds followed by a 1-second pause, whereas in BOLD5000, images are displayed for 1 second and succeeded by a 9-second cross-fixation. Both datasets underwent identical processing to extract the task-related voxel coefficients, specifically within the visual cortex masks. For this particular experiment, our attention was centered on the first three BOLD5000 subjects, which shared 1,000 images with the NSD subjects. This overlap facilitated a direct neural response comparison to the same stimuli across both datasets. The primary objective was to train a decoder using NSD Subject 1, align the common data from BOLD5000 subjects, and then proceed with cross-dataset decoding. For comparative purposes, we implemented the Brain-Diffuser pipeline on the training data (comprising 80% of non-common data) from the BOLD5000 subject to gauge within-dataset and within-subject decoding performances. Subsequently, we employed Ridge Regression to serve as the alignment matrix bridging the neural spaces of BOLD5000 and NSD. After applying this transformation to the BOLD5000 test data, decoding was performed using the decoder trained on NSD Subj01 data. Metrics were computed across the decoded test sets, both within and between datasets. The overarching aim of this experiment was to investigate the feasibility of addressing the intricate task of achieving high-quality cross-dataset and cross-field fMRI data decoding. To further test this cross-dataset experiment, we also leveraged another 3T fMRI dataset, part of the THINGS collection (Hebart et al., 2023). This database, which is richly annotated, consists of 1,854 object concepts representative of the American English language and contains 26,107 manually curated naturalistic object images. In the fMRI study, a representative subset of images from the THINGS database was presented to participants across 12 separate sessions, involving 8,740 unique images of 720 objects. The images were displayed in rapid succession (4.5 seconds), with participants instructed to focus on central fixation. To maintain participant engagement, an oddball detection task was incorporated, requiring responses to occasional artificially-generated images. Additionally, a specific subset of images (100 in total) was repeatedly shown in each session. This task presents a greater challenge in our cross-dataset experiment due to the absence of explicitly common images. To address this issue, we implemented a retrieval system leveraging CLIP embeddings of images and established an alignment dataset consisting of 1,000 images that are semantically similar between the THINGS and NSD datasets. By using these images along with their associated fMRI data, we were able to learn the alignment between the datasets and perform our cross-dataset decoding task.

## 4 Results

### 4.1 Cross-subject decoding experiment

Figures 2and4provide a comparison between stimuli and decoded images from Subj01 (on which the decoding model is trained). These figures also display the aligned activity of all other subjects using Ridge Regression.Figure 5compares fractions of common data used for Ridge Regression-based alignment and different alignment methods. Lastly,Figure 8illustrates each quantitative metric, computed and averaged over the entire test set for each aligned subject (2,5,7). Metrics are expressed as a fraction of the entire dataset, which contains approximately 10 k images per subject (8,859 unique + 982 common across subjects). As common images are necessary, the maximum amount of images that can be included in the alignment process is 952 (termed the “alignment set,” except 30 images left out for visualization purposes), representing around 10% of the dataset. This represents the maximum data that can be incorporated into the procedure, and we experimented with half, a quarter, and a tenth of this data.

**Fig. 4.**
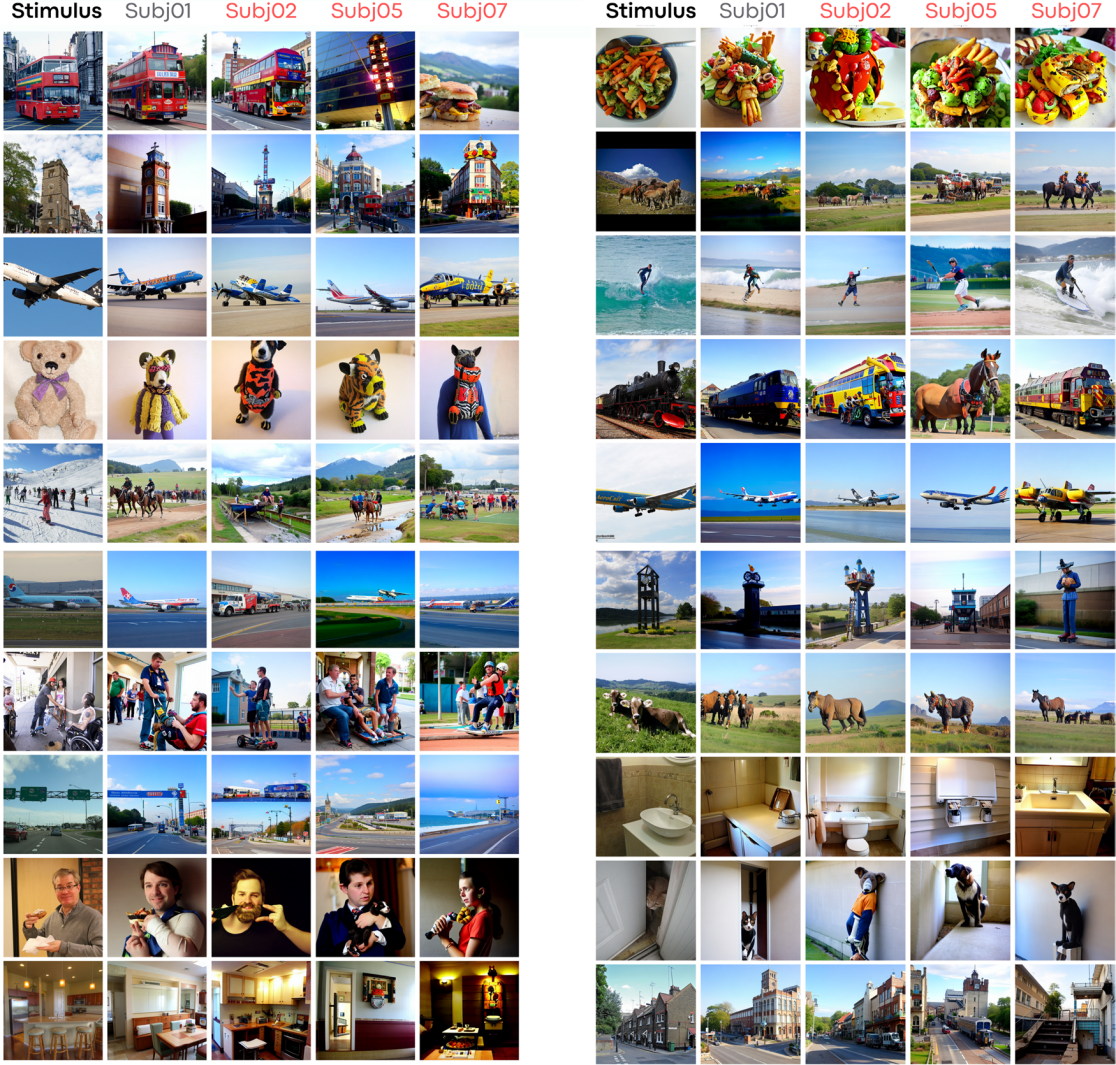
More example results. Format and conventions as inFigure 2.

**Fig. 5.**
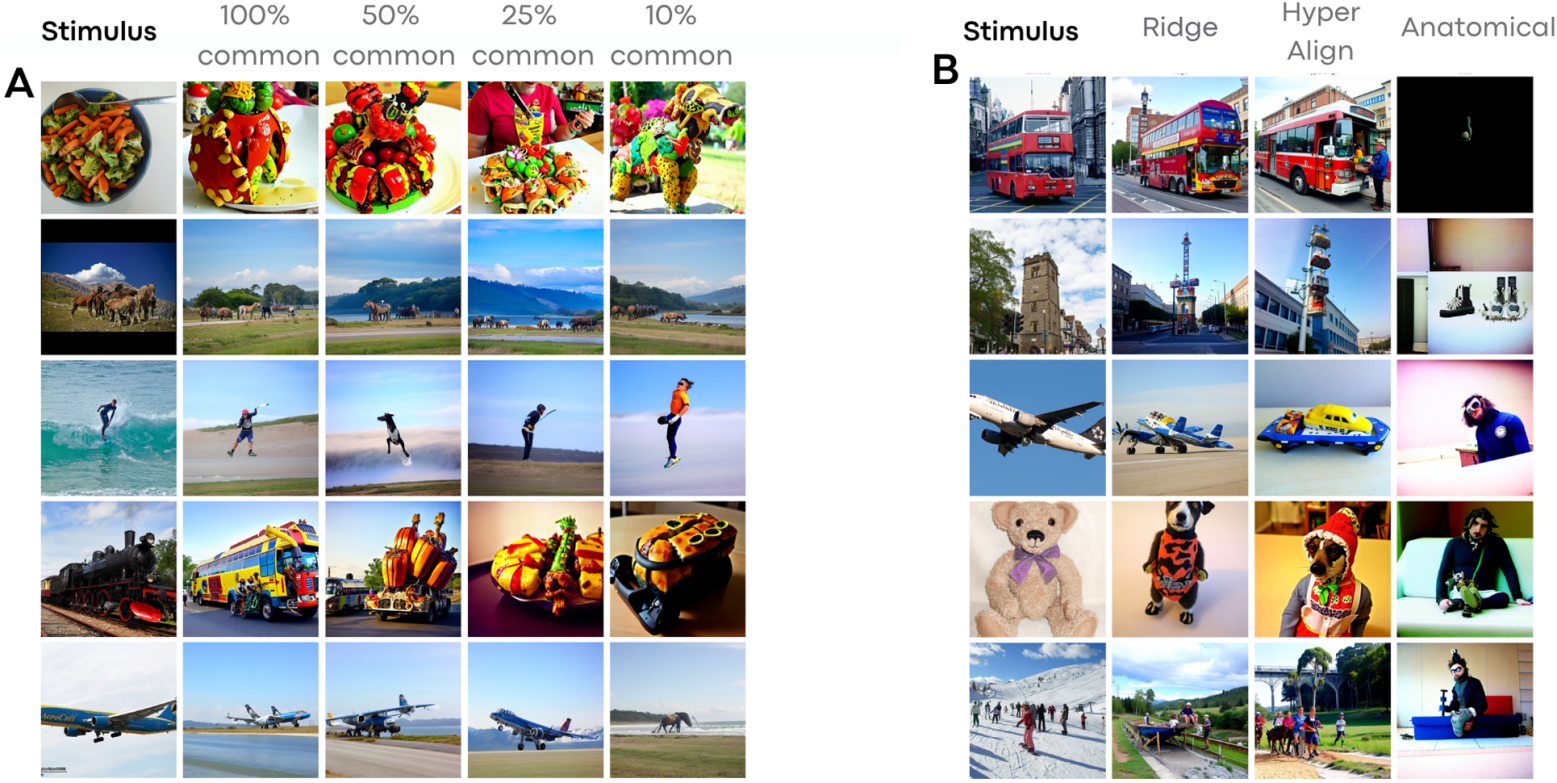
(A) Functional alignment comparison using Ridge Regression across varying fractions of shared data. The “Stimulus” column showcases experimental images, while subsequent columns depict the decoded and aligned activity of Subj02 based on Subj01. (B) A comparison of distinct alignment techniques. The “Stimulus” column again presents the experimental images, with the remaining columns illustrating the decoded activity of Subj02, aligned to Subj01 through various methodologies.

Anatomical alignment fails to yield satisfactory results, demonstrating just above chance performance levels for 2-way classification accuracy and poor performance for low-level metrics such as SSIM and PixCorr. However, Ridge Regression exhibits an increasing performance based on the volume of data used for alignment mapping function learning. This method reaches performance levels comparable with the within-subject decoder in both low-level and high-level metrics, using all the common data (approximately 10% of the entire dataset). An extended version of this figure can be found in theAppendix A, where alignment techniques are compared with within-subject decoders trained on different amounts of data, confirming that performing functional alignment improves performances in decoding when only sparse data are available.

Our findings are encapsulated in the following key points:

#### 4.1.1 Functional alignment’s critical role in fine-grained brain decoding

Our research emphasizes the pivotal role of functional alignment in fine-grained brain decoding. This process, which interprets neural signals to reconstruct perceived images or thoughts, greatly benefits from precise functional alignment of brain activity. Accurate alignment ensures that neural signals are matched correctly to their corresponding brain regions, thus enhancing the decoding accuracy.

#### 4.1.2 Anatomical method’s inefficacy

As corroborated by previous studies (Haxby et al., 2020), our research found that anatomical methods for brain decoding are ineffective. Relying on the physical structure of the brain for alignment and decoding does not deliver the requisite precision for fine-grained decoding tasks. This could be attributed to inherent anatomical variability across different individuals, which may not necessarily align with functional differences. The specialized areas in the brain with functional selectivity can sometimes yield performance above chance levels. However, in most cases, decoded images do not correlate with the stimulus, undermining the reliability of this method for cross-subject brain decoding.

#### 4.1.3 Hyperalignment and ridge regression efficacy

We found that hyperalignment and ridge regression can both be successfully used to perform cross-subject functional alignment and decoding. Our results demonstrate that Ridge Regression-based methods for brain decoding can achieve above-chance performance levels with as little as 1% of the entire dataset. Furthermore, these methods have performances close to baseline levels using around the 10% of the dataset. This crucial finding implies that reliable brain decoding results can be achieved while significantly reducing scan time. This efficiency could be instrumental in making brain decoding research and applications more feasible and cost-effective. These results contribute to our comprehension of the challenges and potential remedies in brain decoding and emphasize the need for additional research to refine these techniques and augment their effectiveness.

#### 4.1.3 Comparable qualitative results from varying training and alignment subjects

**Fig. 6.**
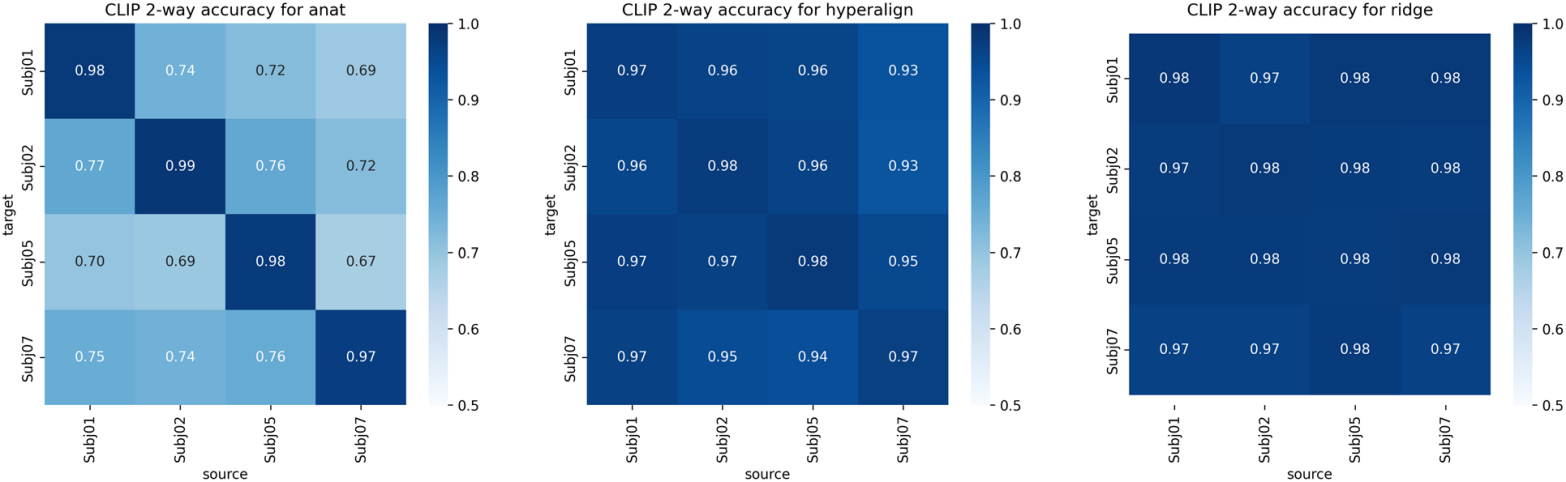
This figure illustrates decoding results with direct CLIP 2-way regressed space, in order to be independent from the actual latent diffusion model used for image generation.

To systematically assess the influence of selecting distinct subjects for model training and alignment, we experimented with multiple combinations. For instance, the decoder was trained on Subject 1, followed by alignment of Subject 2 to this target, and subsequent decoding of Subject 2. This procedure was also executed with the decoder trained on Subject 2, alignment of Subject 1, and decoding of Subject 1. We extended this approach to encompass all potential combinations of our four subjects. As evidenced inFigure 7, the qualitative nature of the decoded images remained consistent irrespective of the subjects chosen for training and alignment. These figures distinctly captured high-level content and foundational shapes across varying subject combinations, yielding analogous visual decoding results.Figure 6presents the direct CLIP 2-way accuracy across all combinations of source and target subjects, utilizing both anatomical and functional alignment. The left side of the figure illustrates that only the diagonal elements yield high decoding accuracy. In contrast, both hyperalignment and ridge regression exhibit off-diagonal values that are comparable to, and on par with, the diagonal elements. This indicates that cross-subject decoding is as effective as within-subject decoding. Within these figures, the diagonal cells represent within-subject decoding, wherein the model undergoes both training and testing on an identical subject. In contrast, off-diagonal cells signify cross-subject decoding, where distinct subjects are employed for training as opposed to alignment and decoding. Despite the variations in quantitative metrics, the visual reconstructions derived from different combinations are qualitatively analogous. This underscores the decoder’s proficiency in generalizing across diverse subjects. The presence of shared neural representations, even amid individual disparities, facilitates precise cross-subject decoding across a spectrum of training and alignment configurations.

**Fig. 7.**
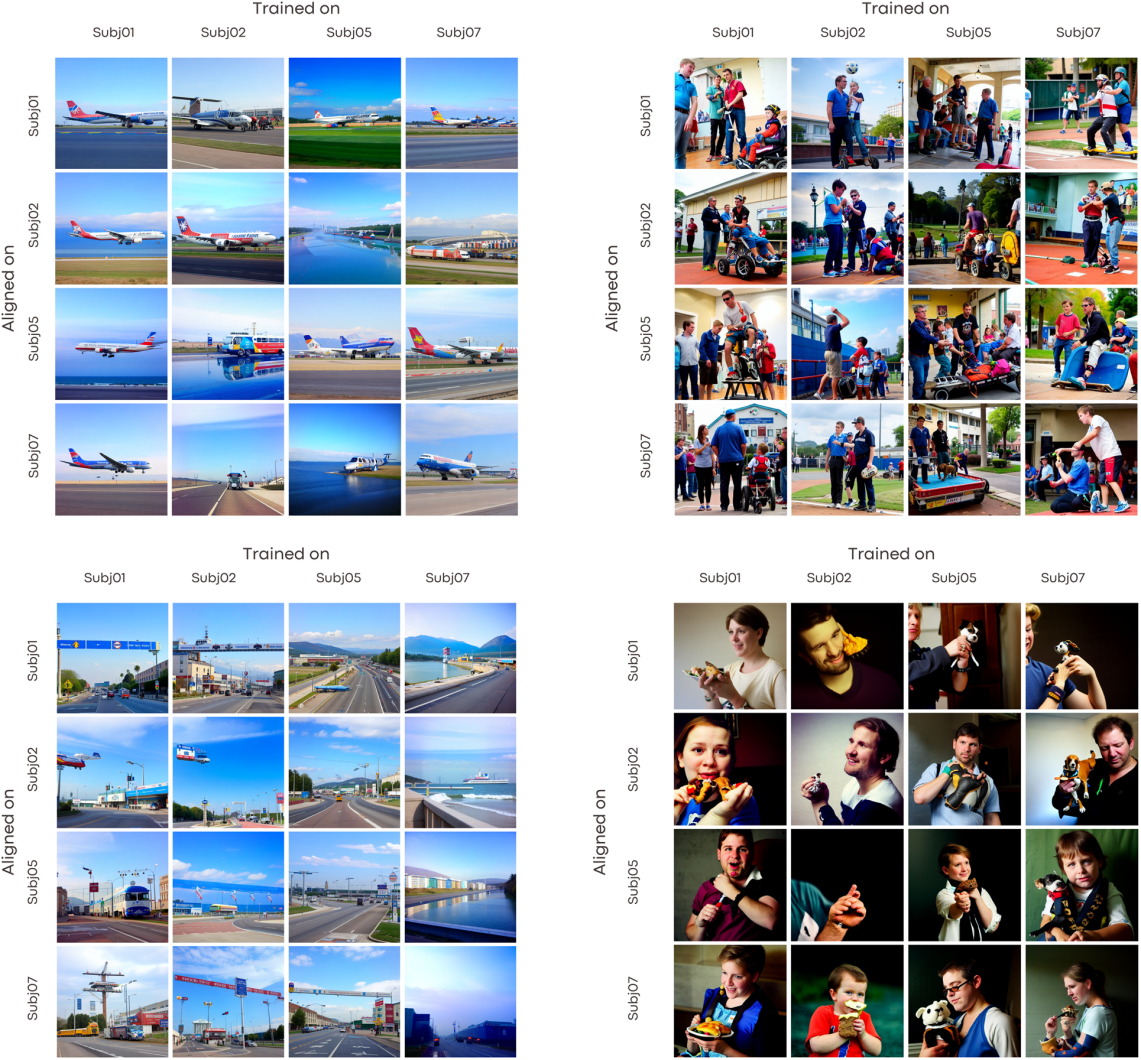
Decoding results from different combinations of subjects used for model training versus alignment. The columns represent decoders trained on individual target subjects. The rows show each remaining subject aligned to the target space of the column subject for decoding.

### 4.2 Cross-dataset decoding experiment

Despite the inherent disparities in acquisition protocols and magnetic field strengths between datasets, we observed successful cross-dataset decoding. Qualitative analysis revealed that reconstructions derived from the aligned BOLD5000 subject were often comparable to those from within-dataset decoding. The same principles apply to the reconstruction of within and cross decoding for the THINGS dataset, highlighting the viability of applying a decoding model across distinct fMRI datasets sharing common stimuli. The primary challenges of cross-dataset application arise from variations in experimental protocols, magnetic field strengths, and even minor details in the images from the THINGS dataset, while maintaining the same semantic content. Nevertheless, our methodology successfully addressed these challenges, achieving effective functional alignment.Figure 9offers a qualitative comparison of reconstructions generated by a model either trained on the same subject or aligned to another subject’s brain activity from the NSD dataset, using its decoder. The alignment technique produced reconstructions that were semantically aligned with the original stimuli, demonstrating a significant advantage of this method. Qualitatively, the performance of decoded image reconstructions was found to be similar when comparing within- and across-dataset decoding. This observation is supported by quantitative metrics, as presented inTables 1and2, which demonstrate that performance on all evaluated parameters using the cross-dataset decoder exceeds chance, thereby validating the feasibility of cross-subject decoding. For the BOLD5000 dataset, a comparison of top-1 accuracy with state-of-the-art decoding methodologies cited inZ. Chen et al. (2022)is also included. Although the decoding approach differs significantly, requiring a pretraining phase on the Human Connectome Project dataset, which includes around 1,000 subjects, followed by fine-tuning on specific BOLD5000 data, it outperforms other methods. The emphasis here is to demonstrate the feasibility of decoding at an individual subject level within the BOLD5000 dataset and through the use of our alignment method and a pretrained decoder on an NSD subject. Comparative metrics indicate that cross-dataset decoding sometimes surpasses within-dataset decoding, suggesting a successful transfer of information despite differences between datasets and subjects. Extending this analysis to the THINGS dataset (Table 2), it was observed that within-subject decoding consistently outperforms cross-dataset decoding, although the latter still achieves performance levels above chance. This further suggests the potential for information transfer. Notably, this task is more challenging than transferring from BOLD to NSD because it relies on semantically similar stimuli rather than identical images.

**Fig. 8.**
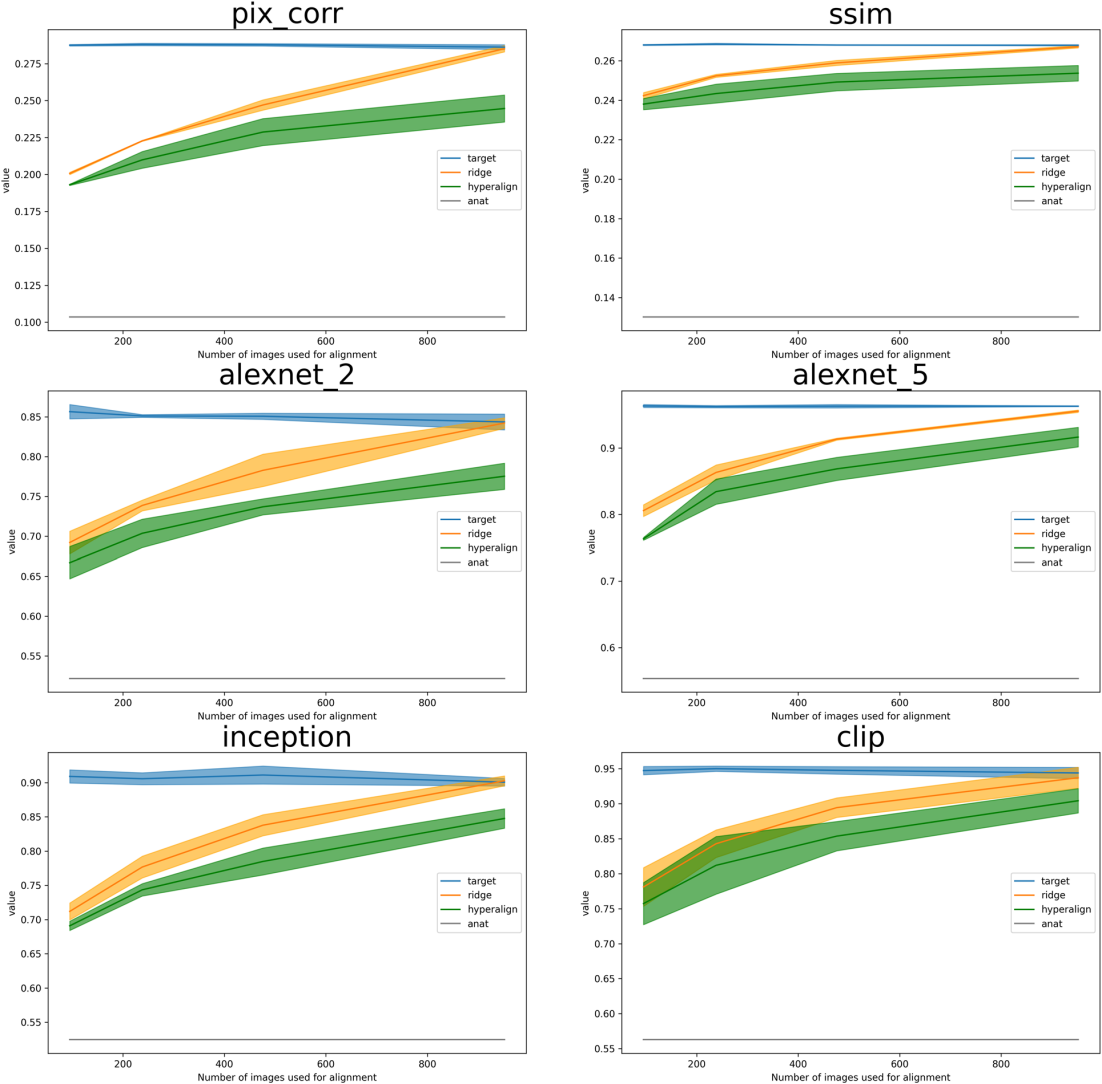
This figure illustrates the performance of alignment methods evaluated using different metrics. Blue lines represent metrics from the target subject’s decoded images, derived from their test set brain activity. Green lines denote the mean and standard deviation (std) of performance on test sets from other subjects, aligned using hyperalignment. Gray lines present results achieved using anatomical alignment, while orange lines display outcomes using Ridge Regression. Remarkably, the Ridge Regression approach yields positive results even when using a tiny fraction of the entire dataset. Furthermore, as this fraction approaches roughly10%of the total set, resulting in 952 images, the performance becomes comparable with that obtained by the within-subject model.

**Fig. 9.**
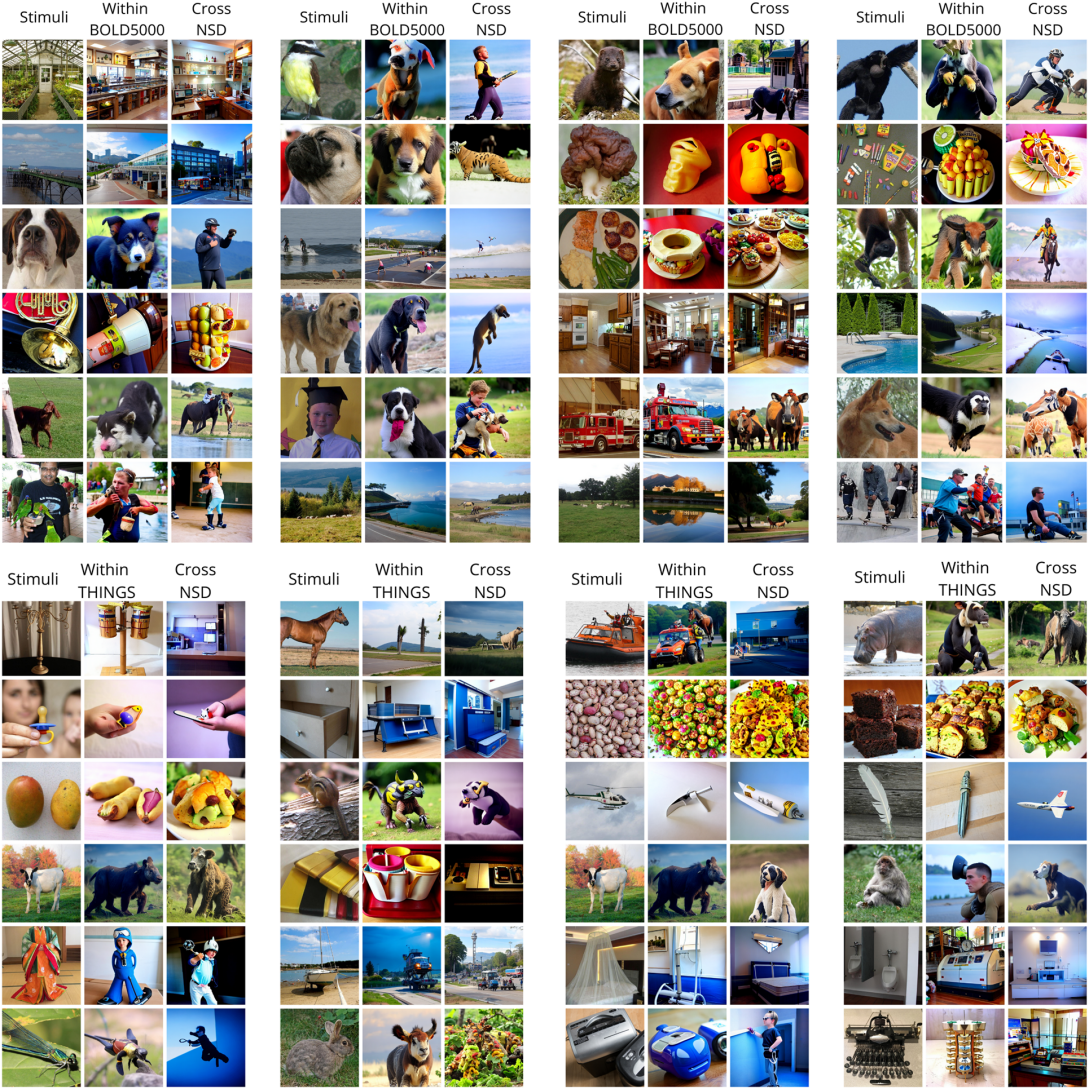
Cross-Dataset qualitative results. Each panel illustrates different random examples from the test set. The first column “Stimuli” shows the original stimulus shown during the experiment. The column “Within BOLD5000” shows reconstruction from a decoding model trained on the BOLD5000 subject. “Cross NSD” column shows results decoded from aligned activity on the NSD dataset and decoded using a decoder trained on the NSD data. Qualitatively, images from this latter column are more semantically similar then the results in the second column. The same happens for the second set of images (panel at the bottom) with the THINGS dataset.

**Table 1.** Expanded table showing quantitative metrics across different datasets and models.

Subj	Pixcorr	SSIM	AlexNet 2nd	AlexNet 5th	Inception	CLIP	Direct CLIP	Top 1 acc
CSI1 (within)	0.1650	0.2201	**0.6009**	**0.7469**	**0.6642**	**0.8126**	0.7104	0.1535
CSI1 (cross)	**0.1736**	**0.2314**	0.5693	0.6691	0.5669	0.7201	**0.7420**	0.115
CSI1 (mind-vis) ( Z. Chen et al., 2022 )	-	-	-	-	-	-	-	**0.3345**
CSI2 (within)	0.1448	0.2114	**0.5669**	**0.7323**	**0.6350**	**0.7761**	0.6909	0.1240
CSI2 (cross)	**0.1560**	**0.2286**	0.5474	0.6593	0.6082	0.6593	**0.7299**	0.0861
CSI2 (mind-vis) ( Z. Chen et al., 2022 )	-	-	-	-	-	-	-	**0.185**
CSI3 (within)	0.1479	0.2221	**0.5693**	**0.7493**	**0.6642**	**0.7591**	0.7250	0.129
CSI3 (cross)	**0.1485**	**0.2321**	0.5304	0.6155	0.5109	0.6715	**0.7323**	0.095
CSI3 (mind-vis) ( Z. Chen et al., 2022 )	-	-	-	-	-	-	-	**0.210**

Bold values indicate superior performance. “Within Data” pertains to results from a decoder trained on BOLD5000 data, while “Cross Data” involves results from reconstructed images using the NSD decoder and aligned BOLD5000 activity.

**Table 2.** Expanded table showing quantitative metrics across different datasets and models.

Subj	Pixcorr	SSIM	AlexNet 2nd	AlexNet 5th	Inception	CLIP	Direct CLIP
sub-01 (within)	**0.1680**	**0.2820**	**0.6633**	**0.775**	**0.64833**	**0.76**	**0.8166**
sub-01 (cross)	0.1496	0.2288	0.53166	0.6000	0.5733	0.6425	0.6524
sub-02 (within)	**0.1642**	**0.2854**	**0.6516**	**0.7916**	**0.65416**	**0.77166**	**0.82999**
sub-02 (cross)	0.14478	0.24148	0.5508	0.62333	0.5608	0.62583	0.6216
sub-03 (within)	**0.15852**	**0.2672**	**0.62583**	**0.73083**	**0.62**	**0.73833**	**0.78083**
sub-03 (cross)	0.15807	0.2282	0.5541666	0.617	0.5425	0.58	0.6233

Bold values indicate superior performance. “Within Data” pertains to results from a decoder trained on THINGS data, while “Cross Data” involves results from reconstructed images using the NSD decoder and aligned THINGS activity.

## 5 Discussion

Our study underscores the intricacies and potential of cross-subject fine-grained brain decoding, a field promising to enhance our understanding of the human brain and cognition.

We identified the criticality of functional alignment for successfully executing brain decoding. This alignment, which maps neural signals to their corresponding brain regions, is vital for accurately decoding neural activity from other individuals using a model pre-trained on separate subject data. This insight holds promise for constructing large studies with a high-accuracy decoding pipeline, subsequently requiring only alignment data acquisition for new subjects. This approach negates the need for an entire experimental reproduction each time, streamlining the process.

Our research reveals the limitations of anatomical methods for brain decoding, which rely on the physical brain structure for alignment and decoding. These methods underperformed due to inherent brain anatomical variability across individuals, which may not align with functional differences. Thus, this study emphasizes the need for functional, not merely anatomical, considerations in decoding studies.

Excitingly, our results suggest significant reductions in scan-time are possible. Ridge Regression-based methods were found to provide reliable brain decoding results with just a fraction of the entire dataset, implying practical implications for brain decoding research feasibility and cost-effectiveness.

Our study also highlights the qualitative similarities in decoded images across subjects. While these images largely match high-level semantic content, intra-subject differences appear minimized. This observation prompts us to consider whether the decoding procedure is fully captured. Given that high-level concepts are generally aligned, we propose a possible brain activity decomposition into*brain activity*=*concept*+*individual perception*. Such a model might only capture the*concept*while treating differences as noise, offering new research directions to explore fine-grained inter-subject differences. This observation might also explain why the metrics for aligned subjects are comparable to or even surpass those of the target subject. The alignment process, which is designed to capture only the visual content common among individuals, could also serve as a form of denoising. This makes it easier for the decoder associated with the target subject to extract information from brain patterns.

Beyond mere conjecture, our results indicate that despite the differences in individual brain structures and functions, it is possible to decode shared neural activity patterns across individuals. This revelation presents compelling prospects for the creation of generalized brain decoding models that can be applied across a diverse range of individuals. Nonetheless, our research also uncovers the constraints inherent in current functional alignment methodologies. As a progression, future investigations might delve into the utilization of more sophisticated models, such as neural networks. These networks are renowned for their capacity to discern intricate, non-linear associations, which could potentially enhance functional alignment.

As we project into the future, several promising avenues for research become evident. These include training models across a spectrum of subjects and devices, potentially culminating in the development of more resilient and universally applicable brain decoding models. For example, using a shared response model, mapping different subjects representations into a common low-dimensional subspace could be a preprocessing step useful to train multisubject large fMRI decoding models. Or, with the advent of larger datasets, future approach could follow the idea described in the work fromBenchetrit et al. (2023)of a shared network with subject-specific alignment layers that at the beginning maps all the subjects into a common representation and then elaborates over this shared space. The inception of novel techniques and methodologies might pave the way for surmounting the existing challenges in brain decoding, ushering in a new era of precise and efficient brain activity interpretation.

In our study, we embarked on a preliminary exploration of this domain through our cross-dataset experiment. The successful cross-dataset decoding achieved between the BOLD5000 or THINGS fMRI and the Natural Scenes Dataset attests to the viability of this approach, even in the face of disparities in acquisition protocols. However, challenges emanate from differences in factors such as magnetic field strength, resolution, stimuli distributions, and more. Designing models that can either account for these variations or remain resilient to them is a formidable task. In forthcoming research, the adoption of intricate techniques, like neural networks equipped with contrastive losses, might aid in discerning invariant feature representations. When trained on a diverse array of fMRI datasets, these methods could effectively identify shared neural patterns, thereby enhancing generalization across varied acquisition specifics. The realm of cross-dataset decoding is burgeoning with potential, offering the prospect of harnessing multiple resources and minimizing the need for fresh data acquisition. The establishment of standardization protocols and shared evaluation benchmarks would significantly bolster this endeavor. Our research underscores the immense potential inherent in cross-dataset brain decoding, paving the way for transcending the constraints of individual datasets.

A potential limitation of our study could be the inherent methodological challenge associated with using the Natural Scenes Dataset, specifically in creating truly independent training and test sets due to the distribution of trials across runs and the computation of GLM’s beta coefficients, which is the standard input for fMRI decoding models in recent literature. This structure could theoretically lead to an overestimation of decoding performance metrics, due to potential information leakage from test trials given that fMRI data are time series and during the computation of beta coefficients all information in a run is used. However, the implementation of advanced analytical methods, such as GLMsingle (Kay et al., 2013;Prince et al., 2022), substantially mitigates this concern given the use of internal and nested cross-validation as well as regularization. This model uses CV to select voxel-wise optimal hemodynamic function, followed by Ridge Regression within the GLM used to obtain regression coefficients, again using a cross-validation procedure. Furthermore, the practice of averaging coefficients for identical stimuli across multiple presentations in different runs further ensures the minimization of possible information leakage between the training and test data. Thus, while the dataset’s structure presents this practical and potential challenge, commonly used methodological precautions and the application of rigorous cross-validation techniques affirm the reliability and validity of our and recent literature findings within the context of this limitation. In our specific case, showcasing model generalization across different subjects and even datasets can be considered robust evidence for true ability of generalization and mitigate concern about potential overfitting.

Furthermore, as we delve into fine-grained brain decoding, addressing potential privacy concerns and ethical implications is paramount. Current research suggests that while certain brain activity aspects can be decoded across subjects, the process is not yet a comprehensive or intrusive “mind-reading” tool. A key finding highlights the disruptive role of attention mechanisms, suggesting that brain decoding is only possible with actively participating, aware subjects.

While our methods currently prevent involuntary or covert “mind reading,” as the field advances, maintaining strong ethical frameworks for brain decoding research becomes even more critical. Informed consent, strict data privacy protocols, and potential societal implications consideration remain key. Decoding brain activity raises broader ethical questions, such as its potential use to enhance communication for individuals with speech or motor impairments or its potential misuse for coercive or manipulative purposes. These critical questions must be confronted by the scientific community and society as we continue to explore brain decoding potential.

## 6 Conclusions

In this research, we detailed a method for brain decoding of visual stimuli across multiple subjects. Our exploration highlighted the importance of functional alignment in decoding neural signals and brought attention to the challenges associated with anatomical methods and complex decoding techniques that might be prone to overfitting.

A key aspect of our study was the application of Ridge Regression-based methods. This approach was effective in decoding neural activity using a subset of the dataset, suggesting potential for reduced scanning durations, possibly nearing 90%. Such reductions could have implications for the efficiency of brain decoding research and its applications.

Our work achieved cross-subject and cross-dataset brain decoding by training the decoding framework on one subject and decoding neural activity of other individuals. This result indicates the presence of shared neural activity patterns, which could be foundational for future generalized brain decoding models. Additionally, we could speculate that results can be explained by a hierarchical structure in the brain’s processing and representation of information, distinguishing brain decoding into concept and perception components. While these findings are promising, we also acknowledge the current limitations in functional alignment methods and see value in exploring other research directions, such as training models across different subjects and devices.

In summary, our study provides insights into the challenges and potential avenues in the realm of cross-subject brain decoding, especially concerning visual stimuli.

## Figures and Tables

**Fig. A1.**
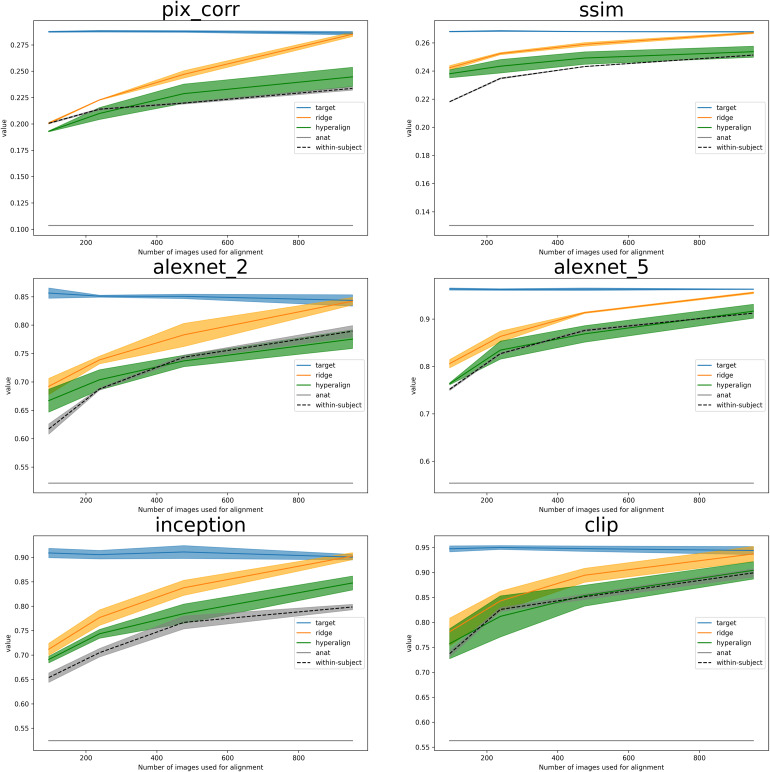
This figure illustrates the performance of alignment methods evaluated using different metrics. Blue lines represent metrics from the target subject’s decoded images, derived from their test set brain activity. Green lines denote the mean and standard deviation (std) of performance on test sets from other subjects, aligned using hyperalignment. Gray lines present results achieved using anatomical alignment, while orange lines display outcomes using Ridge Regression. The black dotted line signifies the performance outcomes of a within-subject decoder that was trained on a small subset of the dataset. Remarkably, the Ridge Regression approach yields positive results even when using a tiny fraction of the entire dataset. Furthermore, as this fraction approaches roughly10%of the total set, resulting in 952 images, the performance becomes comparable with that obtained by the within-subject model.

**Fig. A2.**
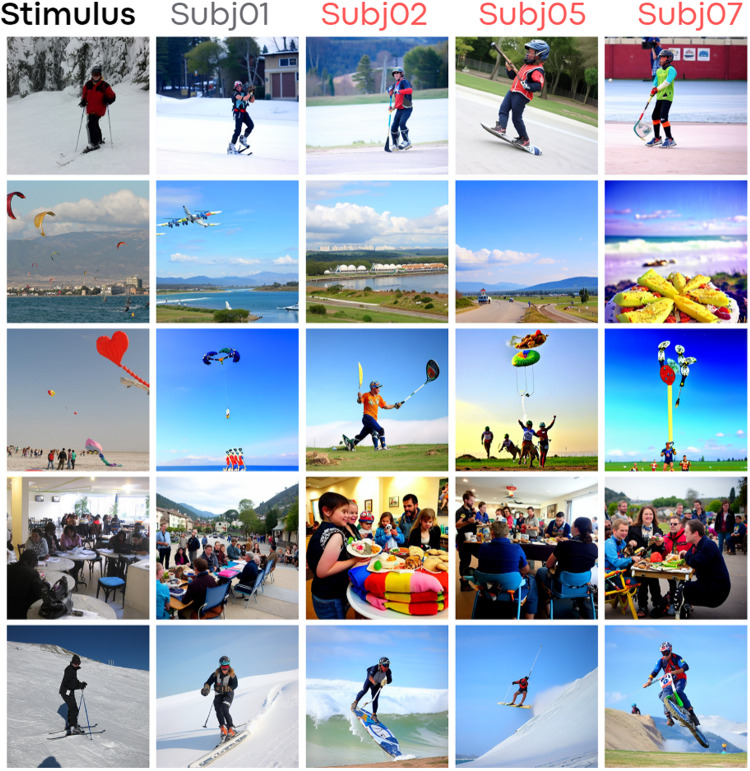
More example results. Format and conventions as inFigure 2.

## Data Availability

Data are publicly available athttps://naturalscenesdataset.org/. As described in the original data paper (Allen et al., 2022), informed written consent was obtained from all subjects, and the experimental protocol was approved by the University of Minnesota Institutional Review Board. The code to perform the cross-subject decoding analysis and reproduce results is available athttps://github.com/matteoferrante/cross-subject-decoding.git

## Appendix A

In this section, we offer further insights and numerical data for all figures presented, to facilitate a comprehensive comparison. In addition to detailed tables, we present findings from an additional experiment. This experiment assesses the efficacy of cross-subject alignment versus within-subject decoding when trained with identical data volumes. It demonstrates that applying cross-subject decoding through a straightforward linear transformation into the target space—where a proficient decoding model is already established—yields superior results compared to direct training on a limited dataset. For this purpose, we replicated the analysis shown inFigure 8from the main manuscript inFigure A1, incorporating an updated dotted line to depict the performance metrics. The latter refer to a decoder trained on the same subject but with varying data quantities. We re-trained the Brain-Diffuser decoding pipeline for all subjects on the same data used for alignment and then measured performances on generated images. The black dotted line in the picture shows an increasing trend with the increase of the dataset size, but performances are always lower than functional alignment.Figure A2provides more examples of decoded images with Ridge regression, whiletables A1–A7show details about decoding performances for each subject and approach.

**Table A1.** Details of the CLIP 2-way accuracy with direct regression for all alignment methods and subjects.

Target	Source				
	Method	Subj01	Subj02	Subj05	Subj07
Subj01	Anat	0.978992	0.742647	0.718487	0.690126
Subj02	Anat	0.766807	0.985294	0.763655	0.718487
Subj05	Anat	0.700630	0.692227	0.977941	0.673319
Subj07	Anat	0.753151	0.743697	0.756303	0.971639
Subj01	hyperalign	0.973740	0.964286	0.961134	0.934874
Subj02	hyperalign	0.957983	0.975840	0.963235	0.929622
Subj05	hyperalign	0.971639	0.965336	0.980042	0.954832
Subj07	hyperalign	0.968487	0.945378	0.943277	0.969538
Subj01	Ridge	0.984244	0.966387	0.981092	0.981092
Subj02	Ridge	0.974790	0.980042	0.976891	0.975840
Subj05	Ridge	0.977941	0.977941	0.975840	0.978992
Subj07	Ridge	0.966387	0.966387	0.977941	0.966387

**Table A2.** Tabular results depicted inFigure 8for Pixel Correlation.

	Method/# of images	95	238	476	952
0	Target	0.287453	0.287965	0.287736	0.286151
1	Anat	0.103591	0.103591	0.103591	0.103591
2	Hyperalignment	0.193022	0.209869	0.228685	0.244612
3	Ridge	0.200703	0.222746	0.247006	0.285059

**Table A3.** Tabular results depicted inFigure 8for SSIM.

	Method/# of images	95	238	476	952
0	Target	0.268018	0.268481	0.267975	0.267864
1	Anat	0.130182	0.130182	0.130182	0.130182
2	Hyperalignment	0.238031	0.243387	0.249211	0.253708
3	Ridge	0.242306	0.252384	0.258937	0.267115

**Table A4.** Tabular results depicted inFigure 8for AlexNet 2nd layer 2-way accuracy.

	Method/# of images	95	238	476	952
0	Target	0.856415	0.850984	0.850645	0.843517
1	Anat	0.521724	0.521724	0.521724	0.521724
2	Hyperalignment	0.667006	0.703666	0.736931	0.775289
3	Ridge	0.692125	0.738629	0.782756	0.842159

**Table A5.** Tabular results depicted inFigure 8for AlexNet 5th layer 2-way accuracy.

	Method/# of images	95	238	476	952
0	Target	0.963340	0.962322	0.963001	0.963001
1	Anat	0.553632	0.553632	0.553632	0.553632
2	Hyperalignment	0.763408	0.834352	0.868635	0.916497
3	Ridge	0.805838	0.863204	0.913442	0.955533

**Table A6.** Tabular results depicted inFigure 8for Inception 2-way accuracy.

	Method/# of images	95	238	476	952
0	Target	0.909029	0.905635	0.911066	0.900543
1	Anat	0.524440	0.524440	0.524440	0.524440
2	Hyperalignment	0.690767	0.743381	0.784793	0.847590
3	Ridge	0.711813	0.776646	0.837746	0.902580

**Table A7.** Tabular results depicted inFigure 8for CLIP 2-way accuracy.

	Method/# of images	95	238	476	952
0	Target	0.947386	0.950102	0.947726	0.943992
1	Anat	0.562797	0.562797	0.562797	0.562797
2	Hyperalignment	0.757298	0.811948	0.853700	0.904277
3	Ridge	0.781059	0.842838	0.894433	0.937203

## References

[b1] Allen , E. J. , St-Yves , G. , Wu , Y. , Breedlove , J. L. , Prince , J. S. , Dowdle , L. T. , Nau , M. , Caron , B. , Pestilli , F. , Charest , I. , Hutchinson , J. B. , Naselaris , T. , & Kay , K. ( 2022 ). A massive 7t fMRI dataset to bridge cognitive neuroscience and artificial intelligence . Nature Neuroscience , 25 ( 1 ), 116 – 126 . 10.1038/s41593-021-00962-x 34916659

[b2] Antonello , R. , Vaidya , A. , & Huth , A. G. ( 2023 ). Scaling laws for language encoding models in fMRI . Thirty-seventh Conference on Neural Information Processing Systems . https://openreview.net/forum?id=2W4LxJbgec PMC11258918 39035676

[b3] Awangga , R. M. , Mengko , T. L. R. , & Utama , N. P. ( 2020 ). A literature review of brain decoding research . IOP Conference Series: Materials Science and Engineering , 830 ( 3 ), 032049 . 10.1088/1757-899x/830/3/032049

[b4] Badrulhisham , F. , Pogatzki-Zahn , E. , Segelcke , D. , Spisak , T. , & Vollert , J. ( 2024 ). Machine learning and artificial intelligence in neuroscience: A primer for researchers . Brain, Behavior, and Immunity , 115 , 470 – 479 . 10.1016/j.bbi.2023.11.005 37972877

[b5] Bazeille , T. , DuPre , E. , Richard , H. , Poline , J.-B. , & Thirion , B. ( 2021 ). An empirical evaluation of functional alignment using inter-subject decoding . NeuroImage , 245 , 118683 . 10.1016/j.neuroimage.2021.118683 34715319 PMC11653789

[b6] Bazeille , T. , Richard , H. , Janati , H. , & Thirion , B. ( 2019 ). Local optimal transport for functional brain template estimation . In A. Chung , J. Gee , P. Yushkevich , & S. Bao (Eds.), IPMI 2019—26th International Conference on Information Processing in Medical Imaging , Hong Kong, China . Springer . 10.1007/978-3-030-20351-1_18

[b7] Benchetrit , Y. , Banville , H. , & King , J.-R. ( 2024 ). Brain decoding: Toward real-time reconstruction of visual perception . The Twelfth International Conference on Learning Representations . https://openreview.net/forum?id=3y1K6buO8c

[b8] Buitinck , L. , Louppe , G. , Blondel , M. , Pedregosa , F. , Mueller , A. , Grisel , O. , Niculae , V. , Prettenhofer , P. , Gramfort , A. , Grobler , J. , Layton , R. , VanderPlas , J. , Joly , A. , Holt , B. , & Varoquaux , G. ( 2013 ). API design for machine learning software: Experiences from the scikit-learn project . In European Conference on Machine Learning and Principles and Practices of Knowledge Discovery in Databases . Prague, Czech Republic , (pp. 108 – 122 ). https://inria.hal.science/hal-00856511

[b9] Busch , E. L. , Slipski , L. , Feilong , M. , Guntupalli , J. S. , di Oleggio Castello , M. V. , Huckins , J. F. , Nastase , S. A. , Gobbini , M. I. , Wager , T. D. , & Haxby , J. V. ( 2021 ). Hybrid hyperalignment: A single high-dimensional model of shared information embedded in cortical patterns of response and functional connectivity . NeuroImage , 233 , 117975 . 10.1016/j.neuroimage.2021.117975 33762217 PMC8273921

[b10] Calhoun , V. D. , Liu , J. , & Adalı , T. ( 2009 ). A review of group ICA for fMRI data and ICA for joint inference of imaging, genetic, and ERP data . NeuroImage , 45 ( 1, Suppl. 1 ), S163 – S172 . 10.1016/j.neuroimage.2008.10.057 19059344 PMC2651152

[b11] Casanova , A. , Careil , M. , Verbeek , J. , Drozdzal , M. , & Romero-Soriano , A. ( 2021 ). Instance-conditioned GAN . In M. Ranzato , A. Beygelzimer , Y. Dauphin , P. S. Liang , J. Wortman Vaughan (Eds.), Advances in Neural Information Processing Systems , (Vol. 34 ). Curran Associates, Inc , pp. 27517 – 27529 . https://proceedings.neurips.cc/paper_files/paper/2021/file/e7ac288b0f2d41445904d071ba37aaff-Paper.pdf

[b12] Caucheteux , C. , Gramfort , A. , & King , J.-R. ( 2022 ). Deep language algorithms predict semantic comprehension from brain activity . Scientific Reports , 12 ( 1 ), 16327 . 10.1038/s41598-022-20460-9 36175483 PMC9522791

[b13] Caucheteux , C. , & King , J.-R. ( 2022 ). Brains and algorithms partially converge in natural language processing . Communications Biology , 5 ( 1 ), 134 . 10.1038/s42003-022-03036-1 35173264 PMC8850612

[b14] Chang , N. , Pyles , J. A. , Marcus , A. , Gupta , A. , Tarr , M. J. , & Aminoff , E. M. ( 2019 ). BOLD5000, a public fMRI dataset while viewing 5000 visual images . Scientific Data , 6 ( 1 ), 49 . 10.1038/s41597-019-0052-3 31061383 PMC6502931

[b15] Chen , P.-H. C. , Chen , J. , Yeshurun , Y. , Hasson , U. , Haxby , J. , & Ramadge , P. J. ( 2015 ). A reduced-dimension fMRI shared response model . In C. Cortes , N. Lawrence , D. Lee , M. Sugiyama , & R. Garnett (Eds.), Advances in Neural Information Processing Systems (Vol. 28 ). Curran Associates, Inc . https://proceedings.neurips.cc/paper_files/paper/2015/file/b3967a0e938dc2a6340e258630febd5a-Paper.pdf

[b16] Chen , Z. , Qing , J. , Xiang , T. , Yue , W. L. , & Zhou , J. H. ( 2022 ). Seeing beyond the brain: Conditional diffusion model with sparse masked modeling for vision decoding . arXiv . https://arxiv.org/abs/2211.06956

[b17] Chen , Z. , Qing , J. , & Zhou , J. H. ( 2023 ). Cinematic mindscapes: High-quality video reconstruction from brain activity . Thirty-seventh Conference on Neural Information Processing Systems . https://openreview.net/forum?id=i913TUOvTK

[b18] Défossez , A. , Caucheteux , C. , Rapin , J. , Kabeli , O. , & King , J.-R. ( 2023 ). Decoding speech perception from non-invasive brain recordings . Nature Machine Intelligence , 5 , 1097 – 1107 . 10.1038/s42256-023-00714-5

[b19] Donahue , J. , & Simonyan , K. ( 2019 ). Large scale adversarial representation learning . In H. Wallach , H. Larochelle , A. Beygelzimer , F. d’Alché-Buc , E. Fox , R. Garnett (Eds.), Advances in Neural Information Processing Systems , (Vol. 32 ). Curran Associates, Inc. https://proceedings.neurips.cc/paper_files/paper/2019/file/18cdf49ea54eec029238fcc95f76ce41-Paper.pdf

[b20] Du , B. , Cheng , X. , Duan , Y. , & Ning , H. ( 2022 ). fMRI brain decoding and its applications in brain and computer interface: A survey . Brain Sciences , 12 ( 2 ), 228 . 10.3390/brainsci12020228 35203991 PMC8869956

[b21] Duan , Y. , Zhou , C. , Wang , Z. , Wang , Y.-K. , & Lin , C.-T. ( 2023 ). DeWave: Discrete encoding of EEG waves for EEG to text translation . In A. Oh , T. Neumann , A. Globerson , K. Saenko , M. Hardt , S. Levine (Eds.), Thirty-Seventh Conference on Neural Information Processing Systems , (Vol. 36 ). Curran Associates, Inc. , pp. 9907 – 9918 . https://proceedings.neurips.cc/paper_files/paper/2023/file/1f2fd23309a5b2d2537d063b29ec1b52-Paper-Conference.pdf

[b22] Ferrante , M. , Boccato , T. , Ozcelik , F. , VanRullen , R. , & Toschi , N. ( 2023 ). Multimodal decoding of human brain activity into images and text . In UniReps: The First Workshop on Unifying Representations in Neural Models . https://openreview.net/forum?id=rGCabZfV3d

[b23] Ferrante , M. , Boccato , T. , & Toschi , N. ( 2023 ). Semantic brain decoding: From fMRI to conceptually similar image reconstruction of visual stimuli . https://arxiv.org/abs/2212.06726

[b24] Gaziv , G. , Beliy , R. , Granot , N. , Hoogi , A. , Strappini , F. , Golan , T. , & Irani , M. ( 2022 ). Self-supervised natural image reconstruction and large-scale semantic classification from brain activity . NeuroImage , 254 , 119121 . 10.1016/j.neuroimage.2022.119121 35342004 PMC9133799

[b25] Gower , J. C. ( 1975 ). Generalized procrustes analysis . Psychometrika , 40 ( 1 ), 33 – 51 . 10.1007/bf02291478

[b26] Haxby , J. V. , Guntupalli , J. S. , Connolly , A. C. , Halchenko , Y. O. , Conroy , B. R. , Gobbini , M. I. , Hanke , M. , & Ramadge , P. J. ( 2011 ). A common, high-dimensional model of the representational space in human ventral temporal cortex . Neuron , 72 ( 2 ), 404 – 416 . 10.1016/j.neuron.2011.08.026 22017997 PMC3201764

[b27] Haxby , J. V. , Guntupalli , J. S. , Nastase , S. A. , & Feilong , M. ( 2020 ). Hyperalignment: Modeling shared information encoded in idiosyncratic cortical topographies . eLife , 9 , e56601 . 10.7554/elife.56601 32484439 PMC7266639

[b28] Hebart , M. N. , Contier , O. , Teichmann , L. , Rockter , A. H. , Zheng , C. Y. , Kidder , A. , Corriveau , A. , Vaziri-Pashkam , M. , & Baker , C. I. ( 2023 ). Things-data, a multimodal collection of large-scale datasets for investigating object representations in human brain and behavior . eLife , 12 , e82580 . 10.7554/elife.82580 36847339 PMC10038662

[b29] Ho , J. K. , Horikawa , T. , Majima , K. , Cheng , F. , & Kamitani , Y. ( 2023 ). Inter-individual deep image reconstruction via hierarchical neural code conversion . NeuroImage , 271 , 120007 . 10.1016/j.neuroimage.2023.120007 36914105

[b30] Horikawa , T. , & Kamitani , Y. ( 2017 ). Generic decoding of seen and imagined objects using hierarchical visual features . Nature Communications , 8 ( 1 ), 15037 . 10.1038/ncomms15037 PMC5458127 28530228

[b31] Huth , A. , Nishimoto , S. , Vu , A. , & Gallant , J. ( 2012 ). A continuous semantic space describes the representation of thousands of object and action categories across the human brain . Neuron , 76 ( 6 ), 1210 – 1224 . 10.1016/j.neuron.2012.10.014 23259955 PMC3556488

[b32] Kay , K. , Rokem , A. , Winawer , J. , Dougherty , R. , & Wandell , B. ( 2013 ). Glmdenoise: A fast, automated technique for denoising task-based fMRI data . Frontiers in Neuroscience , 7 , 247 . 10.3389/fnins.2013.00247 24381539 PMC3865440

[b33] Lange , R. D. , Shivkumar , S. , Chattoraj , A. , & Haefner , R. M. ( 2023 ). Bayesian encoding and decoding as distinct perspectives on neural coding . Nature Neuroscience , 26 ( 12 ), 2063 – 2072 . 10.1038/s41593-023-01458-6 37996525 PMC11003438

[b34] Liu , Y. , Ma , Y. , Zhou , W. , Zhu , G. , & Zheng , N. ( 2023 ). BrainCLIP: Bridging brain and visual-linguistic representation via CLIP for generic natural visual stimulus decoding . https://arxiv.org/abs/2302.12971 10.1109/TMI.2025.3537287 40031248

[b35] Luo , A. F. , Henderson , M. M. , Wehbe , L. , & Tarr , M. J. ( 2023 ). Brain diffusion for visual exploration: Cortical discovery using large scale generative models . Thirty-seventh Conference on Neural Information Processing Systems . https://openreview.net/forum?id=9VqMaSjf7U

[b36] Mai , W. , & Zhang , Z. ( 2023 ). Unibrain: Unify image reconstruction and captioning all in one diffusion model from human brain activity . https://arxiv.org/abs/2308.07428

[b37] Oota , S. R. , Gupta , M. , Bapi , R. S. , Jobard , G. , Alexandre , F. , & Hinaut , X. ( 2023 ). Deep neural networks and brain alignment: Brain encoding and decoding (survey) . https://hal.science/hal-04162064

[b38] Ozcelik , F. , & VanRullen , R. ( 2023 ). Natural scene reconstruction from fMRI signals using generative latent diffusion . Sci Rep 13 , 15666 . 10.1038/s41598-023-42891-8 37731047 PMC10511448

[b39] Prince , J. S. , Charest , I. , Kurzawski , J. W. , Pyles , J. A. , Tarr , M. J. , & Kay , K. N. ( 2022 ). Improving the accuracy of single-trial fMRI response estimates using glmsingle . eLife , 11 , e77599 . 10.7554/elife.77599 36444984 PMC9708069

[b40] Ren , Z. , Li , J. , Xue , X. , Li , X. , Yang , F. , Jiao , Z. , & Gao , X. ( 2021 ). Reconstructing seen image from brain activity by visually-guided cognitive representation and adversarial learning. NeuroImage , 228 , 117602. 10.1016/j.neuroimage.2020.117602 33395572

[b41] Richard , H. , Martin , L. , Pinho , A. L. , Pillow , J. , & Thirion , B. ( 2019 ). Fast shared response model for fMRI data . https://arxiv.org/abs/1909.12537

[b42] Richards , B. A. , Lillicrap , T. P. , Beaudoin , P. , Bengio , Y. , Bogacz , R. , Christensen , A. , Clopath , C. , Costa , R. P. , de Berker , A. , Ganguli , S. , Gillon , C. J. , Hafner , D. , Kepecs , A. , Kriegeskorte , N. , Latham , P. , Lindsay , G. W. , Miller , K. D. , Naud , R. , Pack , C. C. , … Kording , K. P. ( 2019 ). A deep learning framework for neuroscience . Nature Neuroscience , 22 ( 11 ), 1761 – 1770 . 10.1038/s41593-019-0520-2 31659335 PMC7115933

[b43] Scotti , P. S. , Banerjee , A. , Goode , J. , Shabalin , S. , Nguyen , A. , Cohen , E. , Dempster , A. J. , Verlinde , N. , Yundler , E. , Weisberg , D. , Norman , K. , & Abraham , T. M. ( 2023 ). Reconstructing the mind’s eye: fMRI-to-image with contrastive learning and diffusion priors . Thirty-seventh Conference on Neural Information Processing Systems . https://openreview.net/forum?id=rwrblCYb2A

[b44] Shen , G. , Dwivedi , K. , Majima , K. , Horikawa , T. , & Kamitani , Y. ( 2019 ). End-to-end deep image reconstruction from human brain activity . Frontiers in Computational Neuroscience , 13 , 21 . 10.3389/fncom.2019.00021 31031613 PMC6474395

[b45] Takagi , Y. , & Nishimoto , S. ( 2023 ). High-resolution image reconstruction with latent diffusion models from human brain activity . bioRxiv . 10.1101/2022.11.18.517004

[b46] Tang , J. , LeBel , A. , Jain , S. , & Huth , A. G. ( 2023 ) Semantic reconstruction of continuous language from non-invasive brain recordings . Nature Neuroscience , 26 ( 5 ), 858 – 866 . 10.1038/s41593-023-01304-9 37127759 PMC11304553

[b47] VanRullen , R. , & Reddy , L. ( 2019 ). Reconstructing faces from fMRI patterns using deep generative neural networks . Communications Biology , 2 ( 1 ), 193 . 10.1038/s42003-019-0438-y 31123717 PMC6529435

[b48] Vu , M.-A. T. , Adalı , T. , Ba , D. , Buzsáki , G. , Carlson , D. , Heller , K. , Liston , C. , Rudin , C. , Sohal , V. S. , Widge , A. S. , Mayberg , H. S. , Sapiro , G. , & Dzirasa , K. ( 2018 ). A shared vision for machine learning in neuroscience . The Journal of Neuroscience , 38 ( 7 ), 1601 – 1607 . 10.1523/jneurosci.0508-17.2018 29374138 PMC5815449

[b49] Wang , Z. , & Ji , H. ( 2022 ). Open vocabulary electroencephalography-to-text decoding and zero-shot sentiment classification . Proceedings of the AAAI Conference on Artificial Intelligence , 36 , 5350 – 5358 . 10.1609/aaai.v36i5.20472

[b50] Xia , W. , de Charette , R. , Öztireli , C. , & Xue , J.-H. ( 2024 ). DREAM: Visual decoding from reversing human visual system . Proceedings of the IEEE/CVF Winter Conference on Applications of Computer Vision (WACV) .

[b51] Zafar , R. , Malik , A. S. , Kamel , N. , Dass , S. C. , Abdullah , J. M. , Reza , F. , & Abdul Karim , A. H. ( 2015 ). Decoding of visual information from human brain activity: A review of fMRI and EEG studies . Journal of Integrative Neuroscience , 14 ( 02 ), 155 – 168 . 10.1142/s0219635215500089 25939499

